# The Growth and Conidiation of *Purpureocillium lavendulum* Are Co-Regulated by Nitrogen Sources and Histone H3K14 Acetylation

**DOI:** 10.3390/jof9030325

**Published:** 2023-03-06

**Authors:** Ping Tang, Jing-Jing Han, Chen-Chen Zhang, Ping-Ping Tang, Feng-Na Qi, Ke-Qin Zhang, Lian-Ming Liang

**Affiliations:** State Key Laboratory for Conservation and Utilization of Bio-Resources in Yunnan and The Key Laboratory for Southwest Microbial Diversity of the Ministry of Education, Yunnan University, Kunming 650091, China

**Keywords:** nematophagous fungi, conidiation, epigenetic regulation, H3K14, PlGCN5

## Abstract

Plant-parasitic nematodes cause severe economic losses to agriculture. As important biocontrol agents, nematophagous fungi evolved the ability to obtain nitrogen sources from nematodes. However, the impact of nitrogen sources on the growth and development of these fungi is largely unknown. In this study, we aimed to better understand how nitrogen sources could influence vegetative growth and conidiation through epigenetic regulation in the nematophagous fungus, *Purpureocillium lavendulum*. Through nutrition screening, we found a phenomenon of the fungus, limited colony extension with a large amount of conidia production when cultured on PDA media, can be altered by adding ammonia nitrate. Characterized by site-directed mutagenesis, the histone H3K14 acetylation was found to be involved in the alternation. Furthermore, the acetyltransferase PlGCN5 was responsible for H3K14 acetylation. Knockout of Plgcn5 severely diminished conidiation in *P. lavendulum*. Chip-seq showed that H3K14ac distributed in conidiation regulating genes, and genes in the MAPK pathway which may be the downstream targets in the regulation. These findings suggest that histone modification and nitrogen sources coordinated lifestyle regulation in *P. lavendulum*, providing new insight into the mechanism of growth regulation by nutritional signals for the carnivorous fungus.

## 1. Introduction

Global economic losses due to plant-parasitic nematodes amount to hundreds of billions of dollars a year, with an average annual loss of 10–15% of crop yields [1,2]. It is essential to develop green and environmentally friendly methods for the biological control of pathogenic nematodes. Fungi are an integral component of agricultural ecosystems. While many fungi can cause a diverse range of diseases in crop plants [3], some fungi are natural enemies of nematodes that can infect and kill nematodes with various strategies, including forming trapping devices such as rings and nets to capture nematodes, secreting toxins, and conidia germination and penetrating nematode cuticles [4]. For the last strategy, conidia play a vital role in the infection of nematodes [5]. As an important bio-control fungus of nematodes, *Purpureocillium lavendulum* is characterized by its ability to produce a large number of conidia, which are important weapons for the infection of nematodes [6].

As a group of carnivorous fungi, nematophagous fungi sense trophic signals from the environment and their prey, and alter their lifestyle accordingly. Nematodes are the most abundant animals on earth and form a nitrogen pool for their microbial natural enemies like nematophagous fungi [7]. This group of fungi was proposed to be evolved from nonpredatory, cellulolytic or lignin-degrading fungi in nitrogen limited habitats [8]. Thus, the limitation of nitrogen (or lack of prey) may bring about a developmental alteration of nematophagous fungi. Previous studies showed that nitrogen and carbon starvation are important conidiation inducers for fungi [9]. In a carnivorous fungus, the predacious rotiferovorous fungus *Zoophagus insidians*, non-rotifer-fed mycelia produced more conidia than those fed once [10]. Although the gene regulation of fungal conidiation is extensively studied, especially in the model fungi, such and *Neurospora crassa* and *Aspergillus*. spp. [11], how a fungus (especially carnivorous fungi) balances its growth and reproduction by gene regulation in different nutritional environments is still poorly understood.

Posttranslational modifications of histones can regulate diverse biological processes in eukaryotes, including fungal development [12,13]. In *Aspergillus nidulans*, knockout of the histone acetyltransferase GCN5, which catalyzes histone H3K9 and H3K14 acetylation, resulted in a reduction of the hyphal growth rate and the formation of immature and malformed conidia structures [14]. When the GcnE-coding gene (a gcn5 homolog) was deleted in the opportunistic human pathogenic fungus *Aspergillus fumigatus*, the radial growth of fungal colonies and the number of asexual spores were significantly reduced [15]. However, in *P. lavendulum*, the direct biological role of histone modification on asexual development as well as its relationship with nitrogen regulation is largely unknown. In this paper, we present our study on growth and conidiation co-regulated by histone H3K14ac modification and nitrogen resources.

## 2. Materials and Methods

### 2.1. Fungal Strains and Culture Conditions

The nematophagous fungus *P. lavendulum* YMF1.00683 (wild-type) was isolated in Yunnan province, China, and preserved in the Microbial Library of the Germplasm Bank of wild species from Southwest China. The *ku80* strain was constructed previously through high-efficiency homologous recombination [16]. *Agrobacterium tumefaciens* AGL-1 was used for fungal transformation [16]. For the colony phenotype, conidia were diluted into 1 × 10^8^/mL suspension, and 2 μL conidia suspension was inoculated on solid plates for 10 days to observe the growth morphology of each strain on different media. Conidia were counted with a hemocytometer under a light microscope. To compare the effect of changing growth conditions on phenotypes, two media were mainly used, PDA and MM media. The contents of these two media were listed in the Supplementary Materials.

### 2.2. Whole Genome Sequencing of P. lavendulum

In order to facilitate the physiological mechanism study of this fungus from the molecular level, the whole genome of *P. lavendulum* strain YMF 1.00683 was sequenced with the Illumina and Pacbio platforms in the Novogene Biotech Company (Beijing, China). Briefly, genomic DNA was extracted using the Wizard^®^ Genomic DNA Purification Kit (Promega, Madison, WI, USA) using fresh cultured mycelia, followed by RNase treatment. Purified genomic DNA was quantified by Qubit 2.0. A 20K SMRT Bell library for Pacbio sequencing was constructed with a SMRT bell TM Template kit (version 1.0). An Illumina library of 350 bp size was constructed with a NEBNext^®^Ultra™ DNA Library Prep Kit for Illumina (NEB, Ipswich, MA, USA). The genome was sequenced using a combination of the PacBio Sequel and Illumina NovaSeq PE150 sequencing platforms. The PacBio reads were assembled into contigs using CANU and error correction of the PacBio assembly results was performed using the Illumina clean reads. Protein-coding genes were predicted using Maker2. tRNA-scan-SE and Barrnap were used for tRNA rRNA prediction, respectively. Protein-coding genes were annotated through the NR, Swiss-Prot, Pfam, GO, COG and KEGG databases by BLAST. The genome sequence has been deposited in GenBank (accession number: JAQHRD000000000, Bioproject: PRJNA916066, Biosample: SAMN32411341).

### 2.3. Construction of the Histone Mutant Strain

The histone H3 gene of *P. lavendulum* was amplified by primers H3-SbfI-5F/H3-XbaI-5R and inserted into a pEASY-Blunt Zero Cloning plasmid (TransGen, Beijing, China) to obtain a H3-up-T plasmid. The plasmid was then mutated using a Q5 Site-direct Mutagenesis Kit (NEB, Ipswich, MA, USA) and confirmed by sequencing. The mutated H3 gene was PCR amplified by primers H3-SbfI-5F/H3-XbaI-5R as well as an H3 downstream fragment amplified with primers H3-EcoRV-3F/H3-EcoRV-3R. The amplified products were inserted into the digested plasmid pPK2-sur-GFP (kindly provided by Prof. Weiguo Fang, Zhejiang University, China) to construct knockout plasmids [17] by the in-fusion method. Finally, a mutant strain was obtained by *A. tumfacience* mediated-transformation (ATMT) of *P. lavendulum* [16]. Transformants were screened by PCR using primers H3-F/H3-R and confirmed by sequencing.

### 2.4. Western Blotting Analysis

Fungal mycelia were collected by filtration and then were frozen in liquid nitrogen and ground into a fine powder with a mortar and pestle [18]. Protein was extracted with the lysis buffer described previously [19]. Protein concentration was determined by the Bradford assay (Bio-Rad Laboratories, Inc., Hercules, CA, USA), and western blot analysis was performed using standard procedures. Antibodies used in this study include anti-H3K14ac (ab52946) and anti-H3 antibodies (ab1791) (Abcam, Cambridge, MA, USA).

### 2.5. Knockout of Plgcn5 in P. lavendulum

Local BLAST was used to identify the *Plgcn5* gene in the *P. lavendulum* genome with gcn5 as a query of other studied filamentous fungi [20]. The ORF sequence was replaced by the chloropyresulfuron resistance gene (*sur*) using homologous recombination as previously described [16]. Transformants selected from chloropyresulfuron-containing plates were screened by PCR and confirmed by Southern blot analysis (related primers were listed in Supplementary Table S1).

### 2.6. ChIP-Seq Analysis

Fungal conidia (1 × 10^8^) were inoculated into PDB and PDB+(NH_4_)_2_SO_4_ liquid media, respectively, on 9-cm plates with 20mL medium in each plate, stationary cultured at 28 °C for five days. The mycelia were fixed in 1% formaldehyde and terminated with 0.125 M glycine. Routine ChIP experiments with the anti-H3 (acetyl K14) antibody (Ab52946) (Abcam, Cambridge, MA, USA) were conducted as previously described [21]. Immunoprecipitated DNA was sequenced on an Illumina Novaseq6000 platform in Seqhealth Technology Co. LTD (Wuhan, China). Clean reads were mapped to the *P. lavendulum* genome with STAR (2.5.3a), and peak calling was conducted with MACS2. Annotation of peak related genes was performed by bedtools (v2.25.0). GO and KEGG enrichment were analyzed using Kobas (v2.1.2). The raw reads have been deposited in GEO (accession number: GSE222674).

## 3. Results

### 3.1. The Whole Genome Sequence of P. lavendulum

In order to understand the biological regulation of asexual development and characterize the mechanisms for the biological control of nematodes at the molecular level, a high-quality whole genome sequence of the nematophagous fungus *P. lavendulum* is needed. To achieve this, the NGS Illumina and Pacbio platforms were utilized for the de novo genome sequencing of the *P. lavendulum* wild-type strain (YMF1.00683). The Illumina sequencing obtained 3.2 G base pairs of clean data (70×) and Pacbio obtained 2.89 G base pairs of clean data (60×). Twenty-one contigs were assembled with a total length of 46 mega bases. The length of the N50 contig is 4.2 megabases. A total of 8862 genes were predicted and annotated with GO, KOG, KEGG, NR, PHI and CAZy. The mitochondria genome sequence was described in a recently published paper [22].

### 3.2. Effect of Nitrogen Sources on Vegetative Growth of P. lavendulum

**Figure 1:**
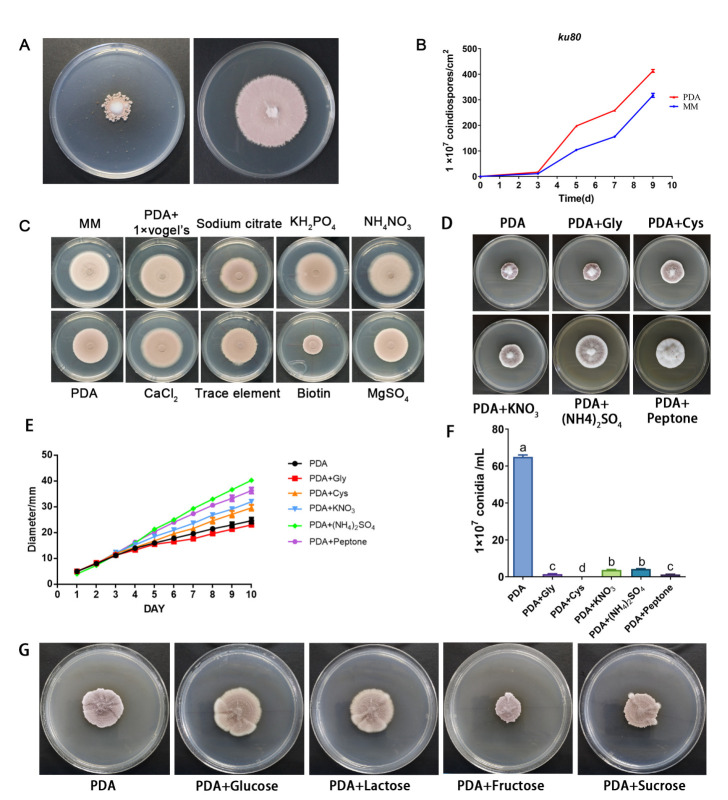
NH_4_NO_3_ affects the growth and conidiation of *P. lavendulum.* (**A**) Comparison of colony morphology of the *ku80* strain cultured on PDA (**left**) and MM (**right**) media for 10 days. (**B**) Conidiation comparison of the *ku80* strain cultured on PDA (**left**) and MM (**right**) media for 10 days duration. (**C**) Comparison of colony morphology of the *ku8*0 strain on MM, PDA, and PDA plus components from MM media. (**D**) Comparison of colony morphology of the *ku80* strain on PDA and PDA with additional nitrogen sources. (**E**) Growth comparison of the *ku80* strain (samples same as (**D**)). (**F**) Conidiation comparison of the *ku80* strain (samples same as (**D**)). Different lowercase letters above columns indicate statistical differences as *p* < 0.05 according to ordinary one-way ANOVA test. (**G**) Comparison of colony morphology of the *ku80* strain on PDA and PDA with additional carbon sources.

To verify how nutrition regulates the development of the nematophagous fungus *P. lavendulum*, we compared the morphology of this fungus cultivated on common laboratory media, PDA, and MM medium. The hypha of the *P. lavendulum* ku80 strain extended slowly on the PDA solid medium, producing small colonies but a large number of conidia. The conidia may also produce satellite colonies around the mother colony (Figure 1A). When cultured on the MM medium, the fungus produced significantly larger colonies compared to those on the PDA medium (Figure 1A), and the number of conidia was also different (Figure 1B). Due to the two media having identical glucose concentrations (2%), we hypothesized that the difference in other nutrient composition in the medium contributed to the observed differences.

In contrast, the colony size of the *ku80* strain was very similar on MM and PDA+1× Vogel’s (the component besides glucose in MM medium) medium (Figure 1C). Adding 0.5× Vogel’s caused an intermediate colony size (Figure S1A,B), indicating that components in Vogel’s could promote the growth ability of *ku80* on the PDA medium. In order to identify the specific nutrient(s) which promoted hyphal growth and reduced conidia production, we separately added each component in Vogel’s solution, which is the main component in MM medium, and compared the growth phenotype in these media. Among the elements added, the growth of the *ku80* strain on PDA+NH_4_NO_3_ and PDA+KH_2_PO_4_ was similar to that on the MM medium (Figure 1C and Figure S1D). Since KH_2_PO_4_ might affect the pH of the medium, we finally identified that NH_4_NO_3_ is the key nutritional factor that can promote *P. lavendulum* growth in the PDA medium. To determine whether ammonium or nitrate was lacking, the two ions were studied separately using (NH_4_)_2_SO_4_ and KNO_3_. It was found that with the increase in concentration, the colony of *ku80* strain increased gradually on both media (Figure 1D and Figure S1C). To further verify this result, common carbon sources and nitrogen sources were added respectively to PDA and MM media, and it was found that nitrogen sources had a great influence on the growth and conidiation of *P. lavendulum*, and that mycelium growth significantly increased (Figure 1D–F). Carbon sources had little effect on conidiation of *P. lavendulum*, with little difference in growth (Figure 1G) and conidiation (Figure S1E). In conclusion, nitrogen sources such as KNO_3_ and (NH_4_)_2_SO_4_ can promote the growth of *P. lavendulum* and can regulate its conidiation.

### 3.3. The Modification of Histone H3K14 Affects the Growth and Conidiation Process of P. lavendulum

**Figure 2:**
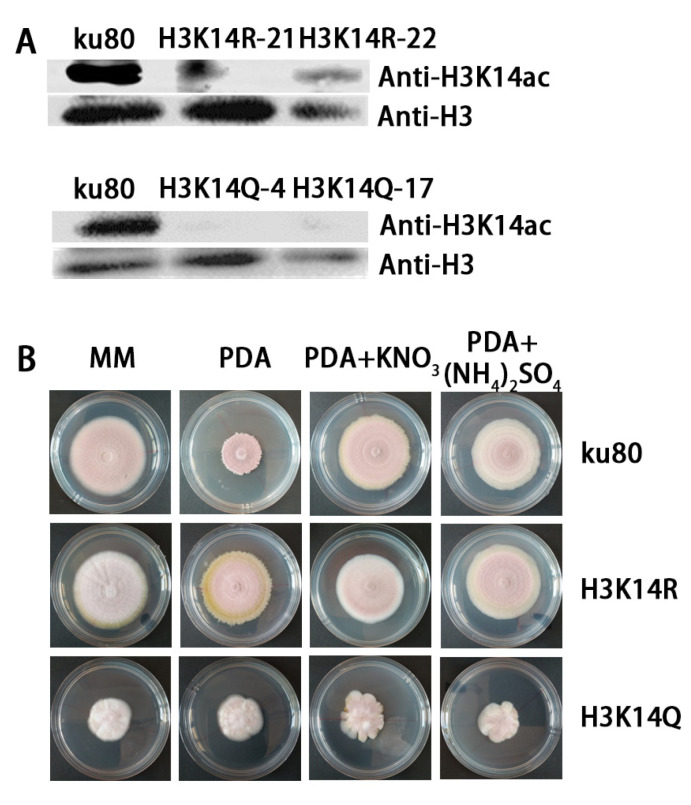
Modification of histone H3K14 affects the growth and conidium of *P. lavendulum*. (**A**) Western blot Q and H3K14Q strains. (**B**) Growth comparison of Histone H3K14R and the H3K14Q mutant, and the *ku80* strain on four media: MM, PDA, PDA+KNO_3_ and PDA+(NH_4_)_2_SO_4_.

To determine whether histone modification affects fungal growth and development, we mutated several tyrosine residues on the histone H3 tail, respectively. The site-directed mutagenesis was verified by sequencing (Figure S2). Among those mutants, we found that the H3K14R strain cultured on PDA plates showed totally different morphology compared to its parent strain (the *ku80* strain). The colonies’ size increased significantly and conidia production decreased significantly, a phenotype that is more similar to the *ku80* strain cultured on MM plates. When the H3K14A strain was growing on MM media, no significant difference was observed compared to colonies on PDA media (Figure 2B). This result revealed that nitrogen regulated fungal development depended on epigenetic regulation of histone H3K14 modification.

H3K14 was then mutated to glutamine (Q), mimicking the acetylation of histone H3K14. Western blot analysis showed that H3K14ac existed in the *ku80* strain but was not detected in the H3K14R and K3K14Q strains (Figure 2A).

The *ku80* and histone mutant strains were compared on MM, PDA, PDA+KNO_3_ and PDA+(NH_4_)_2_SO_4_ mediums respectively (Figure 2B). For *ku80*, the colony on PDA was very different to colonies on the other three media. There was no significant difference in the growth of H3K14R mutant strains on the four media, but conidia production was less abundant than the *ku80* strain. The growth and conidiation of *ku80* were affected by nitrate and ammonium, but the effect was severely weakened by the mutation of histone H3K14 into R (Figure S3). The colony size of the H3K14Q mutant on the four media was similar to that of *ku80*, but the conidiation of the *H3K14Q* mutant was almost inhibited (Figure 2B and Figure S3). Moreover, the H3K14Q strain showed irregular colony margins when cultured on PDA plus the two nitrogen resources. In conclusion, strain *ku80* showed different growth and conidiation on different media with different nitrogen availability. In contrast, the H3K14R and K3K14Q mutants produced fewer conidia, and their colony sizes showed little difference on the four media. These results indicated that the growth and asexual development of *P. lavendulum* by nitrogen stress depends on epigenetic regulation mediated by H3K14 modification.

### 3.4. Knockout of Histone Acetyltransferase GCN5 Attenuated H3K14 Modification and Affected the Growth of P. lavendulum

**Figure 3:**
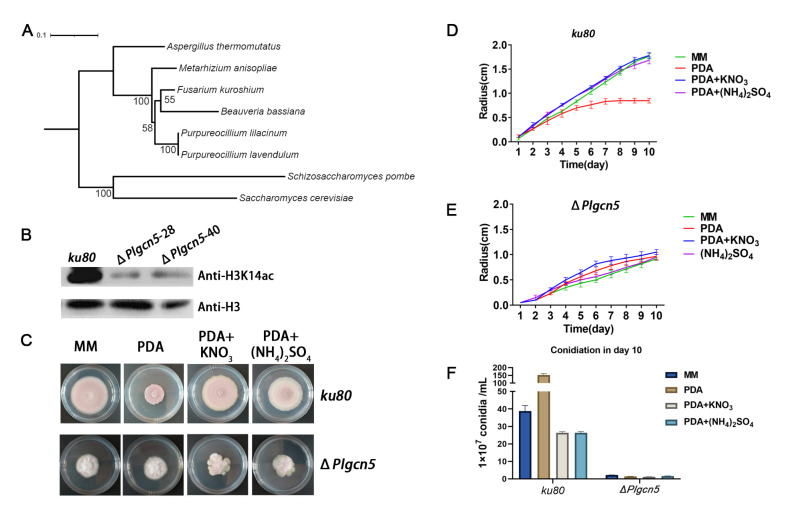
The deletion of histone acetyltransferase *Plgcn5* attenuates H3K14 modification and affects the growth of *P. lavendulum.* (**A**) Phylogenetic analysis of PlGCN5 protein sequences of *P. lavendulum* and other fungi. (**B**) Western blot validation of *Plgcn5* knockout strains using the H3K14ac antibody. (**C**) Comparison of Colony Morphology of the *Plgcn*5 knockout strain and the *ku80* Strain on four media. (**D**,**E**) Growth comparison of the *Plgcn5* knockout strain and the *ku80* strain on four media. (**F**) Conidium comparison of the *Plgcn5* knockout strain and the *ku80* strain on four media.

GCN5 was identified as a histone acetyltransferase to H3K14 acetylation and was known to play an essential role in conidiation in *Aspergillus* species and other fungi [14]. In order to characterize the function of *GCN5* in *P. lavendulum,* we identified the gene sequence of *GCN5* by local BLAST against the whole genome of *P. lavendulum*, using *Aspergillus* GCN5 (XP_026619040.1) as a query, and then carried out phylogenetic analysis of the GCN5 protein sequence of *P. lavendulum* and other fungi by MEGA7 [23]. The results showed that *GCN5* was highly conserved between filamentous ascomycetes, and most likely acetylates the histone H3K14 in *P. lavendulum* (Figure 3A).

The coding region of *Plgcn*5 was knocked out by homologous recombination and confirmed by Southern blot (Figure S4C). Western blot analysis showed that the acetylation level of histone H3K14 decreased in *ΔPlgcn5* (Figure 3B). Compared with *ku80*, *ΔPlgcn5* had a slower growth rate, smaller colonies and sharply reduced conidiation (Figure 3C–E). This phenotype was identical to the H3K14Q mutation. These results indicated that *Plgcn5*-dependent H3K14 acetylation played an important role in the growth and conidiation of this fungus.

### 3.5. Histone H3K14 Acetylation Regulated Genes Involved in the Growth and Conidiation of P. lavendulum

**Figure 4:**
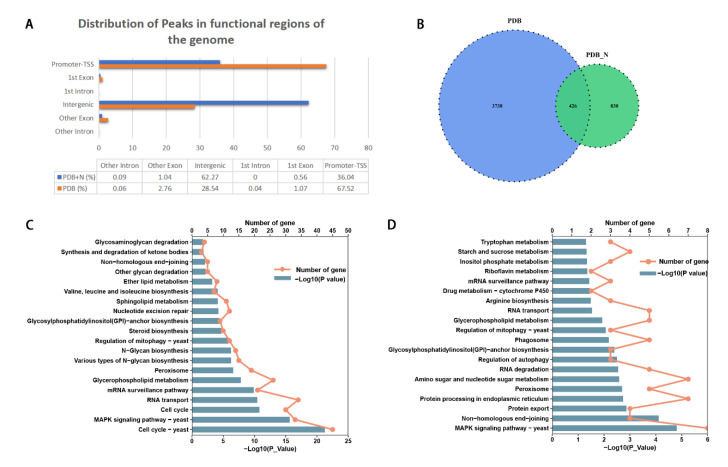
Histone H3K14 acetylation regulates gene expression affecting the growth and conidiation of *P. lavendulum* (**A**) Distribution of peaks in functional regions of the genome. (**B**) a Venn plot comparing gene numbers associated with H3K14 acetylation modification. (**C**,**D**) KEGG enrichment of genes associated with H3K14ac from the PDB (**C**) and PDB with ammonia sulfate (**D**) cultured samples.

In order to further explore the biological role of H3K14ac in *P. lavendulum* and analyze its possible mechanism, ChIP-seq analysis of *P. lavendulum* using an H3K14ac antibody was carried out. Mycelia cultured stationarily on PDB and PDB plus 1% (NH_4_)_2_SO_4_ (PDB+N) liquid media for five days were collected for ChIP-seq analysis. We used Epic2 to call peaks in the IP samples, with the input samples as background. As a result, 4856 and 2331 peaks were identified in the PDB and PDB+N samples, respectively. In the PDB cultured samples, peaks were mainly distributed in promoter-TSS regions (67.52%), and 28.54% of peaks were distributed in intergenic regions. In the PDB plus ammonium sulfate cultured samples, most peaks (62.27%) were distributed in intergenic regions, and only 36.04% of peaks were distributed in promoter-TSS regions (Figure 4A, Figures S5 and S6). This phenomenon revealed that a lack of nitrogen sources in PDB induced a redistribution of H3K14ac modification. In other words, H3K14ac modification is indeed involved in nitrogen dependent gene regulation.

These peaks were distributed in a total of 4994 genes in the whole genome of *P. lavendulum*., among which 4146 genes were in the PDB samples, 1256 genes were in the PDB+N samples, and 426 genes were in both samples (Figure 4B). KEGG enrichment analysis showed that, in the PDB samples, genes associated with H3K14ac were enriched in the cell cycle and the MAPK signaling pathway, etc. (Figure 4C). Genes in the PDB+N samples were enriched in non-homologous end-joining, the MAPK signaling pathway, protein export, protein processing in the endoplasmic reticulum, etc. (Figure 4D). The related genes in MAPK signaling pathway are closely related to the conidiation, development, and pathogenicity of fungi [24,25,26]. We found that 33 genes were enriched in the MAPK signaling pathway in PDB medium cultured samples. These genes were involved in all of the four MAPK pathways, i.e., starvation, high osmolarity, cell wall stress and pheromone response. Only 8 genes were enriched in the MAPK pathway in the PDB+N medium cultured samples, and mainly in the high osmolarity and pheromone pathways (Figure S7).

In a previous study, we characterized several conidiation regulation genes, including the core conidiation regulation genes *PlbrlA*, *PlabaA*, *PlwetA,* and the upstream regulator, *PlfluG* [6]. In ChIP-seq analysis, the above genes were detected to be associated with H3K14ac histones. Meanwhile, compared with the PDB medium supplemented with (NH_4_)_2_SO_4_ cultured samples, we found higher H3K14ac levels associated with these genes in samples cultured with PDB medium. The results suggest a direct regulation of the above genes by H3K14 acetylation (Table S2).

### 3.6. The TOR Signaling Pathway Is Not Directly Involved in the Nitrogen-H3K14ac Regulation

The TOR signaling pathway is an evolutionarily conserved signal transduction system that controls the biological processes in a large number of eukaryotes [27]. One important role is to integrate nutritional signals and regulate cell growth. In ChIP-seq, we found that 19 genes were enriched in the TOR signaling pathway on PDB medium. In order to explore the specific biological role of this pathway, we attempted to knock out the core gene of the pathway, *Pltor*, by homologous recombination. However, no knockout strain could be successfully obtained, suggesting that the TOR pathway is essential in this species.

**Figure 5:**
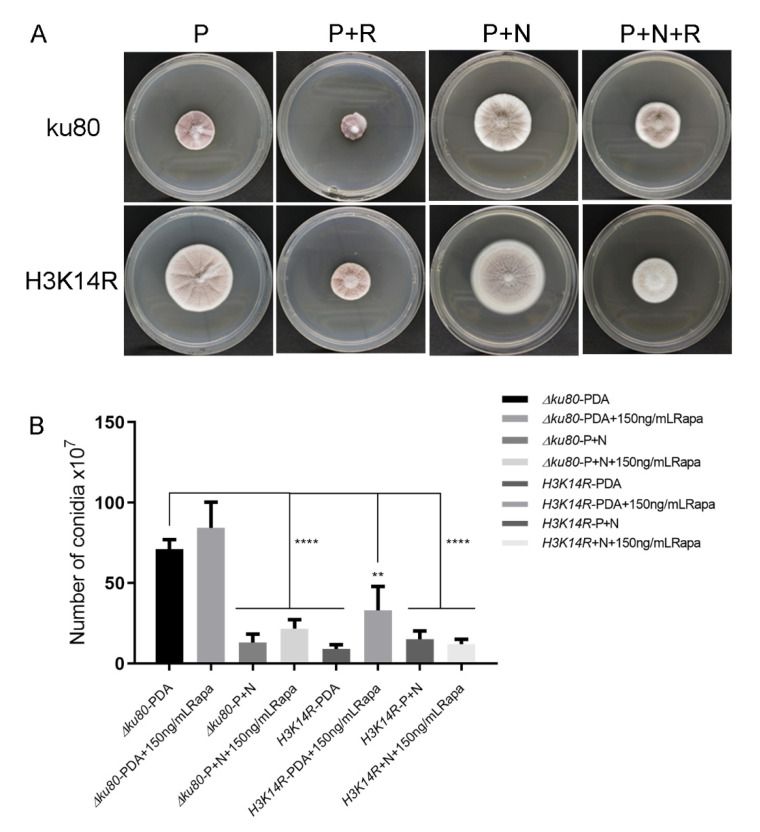
Rapamycin inhibits the growth of *P. lavendulum*. (**A**) Comparison of colony morphology of *ku80* and *H3K14R* strains in PDA (P), PDA + rapamycin (P+R), PDA + (NH_4_)_2_SO_4_ (P+N) and PDA + (NH_4_)_2_SO_4_ + Rapamycin (P+N+R). (**B**) Conidiation comparison of *ku80* and *H3K14R* strains on the four media listed in (A). **, *p* < 0.01; ****, *p* < 0.0001.

The TORC1 complex is known to be inhibited by rapamycin [28]. Therefore, we cultured the *ku80* and histone modified mutant strains in media containing rapamycin (150 ng/mL). All strains grew more slowly, and their colonies were significantly diminished in the rapamycin-containing medium than in the original medium (i.e., PDA + Rapamycin vs. PDA; PDA + (NH_4_)_2_SO4 + Rapamycin vs. PDA + (NH_4_)_2_SO4). However, adding rapamycin did not change the phenomenon that H3K14R grew faster than the ku80 strain in nitrogen poor media, but not in a nitrogen rich media (Figure 5A,B). In addition, adding rapamycin did not change conidiation significantly in any of the three strains (Figure 5C). The above results showed that the TOR signaling pathway affected hyphal growth as rapamycin inhibited all strains on all type of media, suggested this pathway may act on the upstream of the nitrogen-epigenetic regulation of mycelia growth.

## 4. Discussion

As opportunistic carnivorous fungi, nematophagous fungi such as *P. lavendulum* often have sophisticated regulations between hyphal growth and reproduction in response to nitrogen sources, including from their nematode prey. Initiation of asexual reproduction development (conidiation) can be induced by a variety of environmental factors, including mainly environmental stresses such as limited carbon and nitrogen sources [29]. How these environmental signals induce conidiation is poorly understood. In a previous study, we reported the central regulation genes of conidiation, *brlA*-*abaA*-*wetA*, in *P. lavendulum* [6]. The molecular mechanism of nitrogen starvation induced fungal life cycle transformation from the perspective of epigenetic regulation is poorly understood. In the present study, we demonstrated that nitrogen sources and H3K14 modification play key roles in regulating the growth and conidiation of *P. lavendulum*.

Nitrogen is an essential component of amino acids and nucleic acids. Therefore, sufficient nitrogen sources are very important for the biological processes of all organisms. For nematophagous fungi, one solution for obtaining the limited supply of nitrogen is to capture/infect and consume nematodes that are abundant in the soil and other environments where decomposed organic matter is plentiful [30]. Unlike nematode-trapping fungi, which can produce special trapping devices to capture nematodes, the conidia are the weapons to infect nematodes. Thus, producing a large number of conidia under nitrate starvation is a survival and dispersal strategy, to initiate living in a new habitat or to infect nematodes. In the present study, we found that when cultured on PDA media, which is composed mainly of starch and glucose, with a high carbon-to-nitrogen (C/N) ratio, *P. lavendulum* had a limited colony size and produced large amounts of conidia. We could easily observe that, around the central colony, satellite colonies were grown from newly produced conidia (Figure 1A). On the other hand, when cultured on MM media, the hypha extended much faster and resulted in only one large colony in the plate (Figure 1A). We found that the NH_4_NO_3_ in MM medium is the key nitrogen source related to the above phenotype changing. In addition, nitrate and ammonium were both found to affect the growth and conidiation of the *ku80* strain when added to PDA medium. Our results are consistent with studies on other fungi. Müller et. al. had shown that nitrogen starvation induces conidiation in Neurospora crassa [31]. In another study, a high C/N ratio provided the highest level of spores in the fungus *Penicillium camemberti* [32], and in the important biocontrol agent for *Penicillium oxalicum* [33].

The nitrogen starvation induced conidiation depends on epigenetic regulation. Epigenetic regulation related to the maintenance of the chromosome structure, via DNA, RNA and/or histone modification, etc., often regulates developmental processes in multicellular organisms, including filamentous fungi [34]. It has been shown that the acetylation of histone H3K14 has significant effects on fungal growth, conidiation or toxicity [35,36]. In this study, we found that the phenotype of H3K14 point mutant strains was significantly different from that of the *ku80* strain. The restricted growth and increased conidiation induced by nitrogen starvation on the PDA medium were altered when H3K14 was mutated to arginine. H3K14R mutants, mimicking non-H3K14 acetylation, were more like ku80 cultured on MM media. This result suggests that nitrogen starvation regulation relies on H3K14 acetylation. In *Aspergillus*, the function of *GcnE (GCN5)* is necessary for the acetylation of the promoter of the main regulator of histone H3K9/K14 in conidia, and the conidia defect occurs in *GCN5* knockout strains (Cánovas, Marcos et al. 2014). Loss of *GCN5* in *Beauveria bassiana* leads to low acetylation of H3 at the K9/14/18/27 site, which results in reduced conidiation capacity of the strain and serious defects in colony growth and conidial heat resistance [36]. Compared with the *ku80* strain, GCN5 knockout strains grew much slower and produced few conidia. To some extent, this phenotype was previously reported in *Aspergillus* spp., as *gcn5* KO mutants did not produce conidia, although the colony size was similar to the wild type strain [14]. On the other hand, H3K14Q mutants mimicking H3K14 acetylation showed similar growth rates and conidiation, suggesting that global acetylation or deacetylation of histone H3K14 sites can affect vegetative growth and conidiation of *P. lavendulum*, while the specific gene sets regulated by H3K14 modification in the nitrogen starvation regulation should be characterized.

Through ChIP-seq analysis, nitrogen starvation changed H3K14 acetylation modification patterns in the entire genome of *P. lavendulum*. The acetylation of histone H3K14 regulates the conidiation genes of *P. lavendulum,* including the core regulation genes *brlA, abaA, wetA,* and *fluG* (Table S2). Many pathways are regulated by H3K14ac, especially the MAPK signaling pathway. In other fungi, there are also many MAPK pathway-related genes involved in conidia production. In the rice blast fungus *Magnaporthe grisea*, knockout of mps1, a yeast Slt2 mitogen-activated protein kinase homolog, reduced sporulation and fertility and resulted in the non-function of appressoria. Mutating the *OSM1* gene of *M. grisea,* a hog1 homolog in yeast, led to 10-fold reduced conidiation [37]. In the anthracnose fungus *Colletotrichum lagenarium*, the *M. grisea* PMK1 homolog, Cmk1, is required for conidiation and conidial germination [38]. It is speculated that the MAPK pathway is an essential pathway related to conidiation of *P. lavendulum*, and many genes in the pathway are epigenetically regulated by H3K14ac modification, including *ste11, sst2* and *bem1*, etc. The exact biological role of these genes and their epigenetic regulation need to be further assessed.

The TOR (target of rapamycin) Ser/Thr protein kinase is the central component of a eukaryotic signaling pathway that regulates growth [39]. TOR kinases control gene expression and cell differentiation in fungi through nutrient signaling [40]. When cultured on media with rapamycin, all the *P. lavendulum* strains grew slowly and their colonies became smaller than those grown on media without rapamycin (Figure 5A). Adding rapamycin promoted conidia production to some extent in the samples of the first three groups (Figure 5B). It was confirmed again that nitrogen sources can promote the growth of mycelia and delay conidiation, and suggested that the TOR signaling pathway plays an essential role in *P. lavendulum*. This pathway is involved in hyphal growth, asexual development and conidiation, but does not seem to be involved in the nitrogen-epigenetic regulation route.

In conclusion, we reveal that nitrogen induced histone H3K14ac modification plays a crucial role in regulating mycelial growth and conidiation of *P. lavendulum*. The acetyltransferase *GCN5* was responsible for H3K14ac modification and the MAPK pathway was regulated by H3K14ac. These findings could provide new insights into the fungal life cycle regulation of a carnivorous fungus and lay the groundwork for developing bio-control agents using the the nematophagous fungus *P. lavendulum*.

## Data Availability

The genome sequence of *P. lavendulum* has been deposited in GenBank (accession number: JAQHRD000000000, Bioproject: PRJNA916066, Biosample: SAMN32411341). The raw reads of the ChIP-seq experiment have been deposited in GEO (accession number: GSE222674).

## Supplementary Materials

The following supporting information can be downloaded at: https://www.mdpi.com/article/10.3390/jof9030325/s1, Media formulations, Table S1: Primers used in this study; Table S2: Peak statistics in conidiation related genes in ChIP-seq, Figure S1: Growth and conidiation of P. lavendulum in various conditions; Figure S2: Sequencing H3 genes to confirmation of H3K14R and H3K14Q mutation; Figure S3: Growth and conidiation of ku80 and two mutants on four different media; Figure S4: PlGCN5 knockout validation; Figure S5: The heatmap of peak distribution around the TSS in samples cultured in PDB media; Figure S6: The heatmap of peak distribution around the TSS in samples cultured in PDB plus ammonium sulfate media; Figure S7: H3K14ac is enriched in the MAPK pathway.
