# Supplementary material for: The Growth and Conidiation of Purpureocillium lavendulum Are Co-Regulated by Nitrogen Sources and Histone H3K14 Acetylation
Source: J Fungi (Basel). 2023 Mar 6;9(3):325. doi: 10.3390/jof9030325 (PMC10054409; doi:10.3390/jof9030325)
Supplement: Supplementary file 1 [file jof-09-00325-s001.zip › jof-2237960-supplementary.pdf]

Supplementary materials

The Growth and Conidiation of *Purpureocillium lavendulum* Are Regulated by Nitrogen Source and Histone H3K14 Acetylation

Ping Tang [†], Jing-Jing Han [†], Chen-Chen Zhang, Feng-Na Qi, Ping-Ping Tang, Ke-Qin Zhang, Lian-Ming Liang *

State Key Laboratory for Conservation and Utilization of Bio-Resources in Yunnan and The Key Laboratory for Southwest Microbial Diversity of the Ministry of Education, Yunnan University, Kunming, 650091, China.

[†] These authors contributed equally to this work.

*Correspondence: lianglm@ynu.edu.cn (L-ML); Tel: +86 871 65033805;

Fax: +86 871 65034838;

Media formulations：

PDA: glucose 2%, potato 200g/L (boiled for 30min, filtered with gauze), agar 2%.

MM: Vogel's 20ml/L, glucose 20g/L, agar 20g/L.

50×Vogel' s : Sodium citrate·$2H_2O$ 12.5%(w/v)，KNO_3 12.6%, KH_2PO_4 8%(w/v)，$MgSO_4 \cdot 7H_2O$ 1% (w/v), $CaCl_2 \cdot 2H_2O$ 0.5% (w/v), Trace element 5mL /L, Biotin Solution(0.1mg /L) 2.5mL /L, Chloroform 2ml/L (As a preservative)

Trace element(w/v)：Citric acid.H_2O 5%, $ZnSO_4.7H_2O$ 5%, $Fe(NH_4)_2(SO_4)_2.6H_2O$ 1%, $CuSO_4.5H_2O$ 0.25%, $MnSO_4.H_2O$ 0.05%, H_3BO_3 0.05%, $Na_2MoO_4.2H_2O$ 0.05%.

Table S1. Primers used in this study

Primer Name	Sequence（5’-3’）	Fragment size (bp)	Function
**GCN5-5f**	GGCCAGTGCCAAGCTTGCATGCCTGCAGGGAA ATCTCCGCGAGCGAATC	1228bp	Amplification of upstream fragment of GCN5, The underline represents the homologous sequence with the restriction site of plasmid, and the thickening sequence is the restriction site.
**GCN5-5r**	GGCGTTGGcACAGATCTAGTcTCTAGACGTGTC AACCTCACCTACCCATC		
**GCN5-3f**	ACGGGAATTGCATGCTCTCAcACTAGTGATCC AGAGCTGc ACATACAccG	1289bp	Amplification of downstream fragment of GCN5

**GCN5-3r**	TAATCGACCGACGGAATTGAGGATATCGGAGC AGGGCATC ATCATTCG		
**GCN5-F**	CGAACCCGACGAGGAACT	3210bp	Verify that GCN5 is knocked out
**GCN5-R**	GCCTGCGTTGTGTTTAGCC		
**GFP-F**	CACCTTGATGCCGTTCTT	750bp	Random insertion validation of GCN5 knockout strain
**GFP-R**	AcCCTTTGGCTCGCTTA		
**GCN5-T-F**	TCCGACGCAAACCTGATG	604bp	Preparation of Southern blot probe
**GCN5-T-R**	AGTCCGTTCCTCCCCACA		
**H3-5F**	CCGACGAAGTATGGTAGACACC	2275 bp	Upstream segment of histone H3 gene connected to pEASYH3-5R CGAGGACGTAGCCAATCAAAA Blunt Zero Cloning
**H3-5R**	CGAGGACGTAGCCAATCAAAA		
**H3-SbfI-5F**	GGCCAGTGCCAAGCTTGCATGCCTGCAGGCCG ACGAAGTATGGTAGACACC	2275 bp	Amplification of upstream fragment of histone H3 gene
**H3-XbaI-5R**	GGCGTTGGCACAGATCTAGTCTCTAGAC GAGGACGTAGCCAATCAAAA		
**H3-EcoRV-3F**	CATGCTCTCACACTAGTGACTGATATC GAAGAGTCGCATCGTCAAACT	1022 bp	Amplification of downstream fragment of histone H3 gene
**H3-EcoRV-3R**	TAATCGACCGACGGAATTGAGGATATC GGTGGTGGTCAACAAAGGGT		
**PEASY-F**	TGCTAAGGCGCACGACCC	942bp	Sequencing primers to find the correct mutant plasmid
**PEASY-R**	ACTCGAATAAGCCTTTG		
**H3-F**	ACCAGTGCATCAACTCGTAT	1443bp	Genome Validation for Transformation of Mutant Vector into P. lavendulum
**H3-R**	GCTTGGTGATGCCCTGAAT		
**brlA-CDS-R**	CTCACCATGTTGACGGTTGTGAAATCGTCGCC CCGCTTCTC	1163bp	Amplification of brlA-CDS can be used as a linker between brlA-CDS and GFP
**brlA-CDS-F**	CCAAGCTTGCATGCCATGCAGTTCGAATCCGA CTTT		

**GFP-F**	ACAACCGTCAACATGGTGAGC	946bp	Amplification of GFP fragment as a linker primer between brlA-CDS fragment and GFP fragment
**GFP-R**	AGATCTAGTCTCTAGGTTGATAATGGGAATTGATTA		
**brlA-GFP-3F**	ATGCTCTCACACTAGGACACACATTGCCCGGCGTCT	1379bp	Amplification of downstream homologous arms
**brlA-GFP-3R**	TCGCGGCCGCGGATCCATCCATCCAGTCCAGACTCC		
**GFPxiaYZ-F**	CAGTGCATCAACTCGTATAG	1662bp	Sequencing primers to verify whether the downstream fragment was successfully connected and genome validation of the mutant transformed into P. lavendulum
**GFPxiaYZ-R**	ACTGGAAAGCGGGCAGTGAG		
**brlA-GFP-ShangYZ-F**	CTCTTCGCTATTACGCCAGC	2121bp	Sequencing primers to verify whether the upstream fragment was successfully connected and genome validation of the mutant transformed into P. lavendulum
**brlA-GFP-ShangYZ-R**	CATGTTTGCCGCCATCGGAG		
**second-TOR3-F**	aaacactgatagtttAGTTTTGGGGCTGGAGAGT	743bp	The downstream homologous arm was amplified to verify whether the downstream fragment was successfully connected.
**second-TOR3-R**	actgctggcctctagGCGGCTTTATTAGCATTGTTC		
**second-TOR5-F**	ggcattatacactagGTCGGCGGCTGAGATTTAG	977bp	The upstream homologous arm was amplified to verify whether the downstream fragment was successfully connected.
**second-TOR5-R**	atggacgagctgtacTGCTTGTCTGCGTGCTGTT		
**YZ-tublin-F**	GCACAATCATCGCAAACCG	1165bp	Validation of genomic tublin after vector plasmid transformed into P. lavendulum
**YZ-tublin-R**	GCCCCGACAACTTCGTCTT		
**tor-geneYZ-tublin-F**	TTGTGGTCCCCAAAGTA	830bp	Validation of TOR vector plasmid transformed into genome tublin of P. lavendulum

**tor-geneYZ-tublin-R**	AGCGACATAAGCATCCC

Table S2. Peak statistics in conidiation related genes in ChIP-seq

**Order**	**Gene**	**In P. lavendulum**	**ChIP-seq Peaks**		**Function**
			PDB	PDB-N	
**1**	*PlbrlA*	Contig6.18	3	0	Production conidia core, spore germination
**2**	*PlabaA*	Contig3.44	8	1	Determines the differentiation of conidiophores
**3**	*PlwetA*	Contig3.72	2	0	Conidia cell wall integrity, conidia maturation, regulation of trehalose production
**4**	*PlflbA*	Contig1.16	32	5	Normal conidiation, conidiation decreased after knockout
**5**	*PlfluG*	Contig4.91	5	0	Normal conidiation and delayed conidiation after knockout

Figures

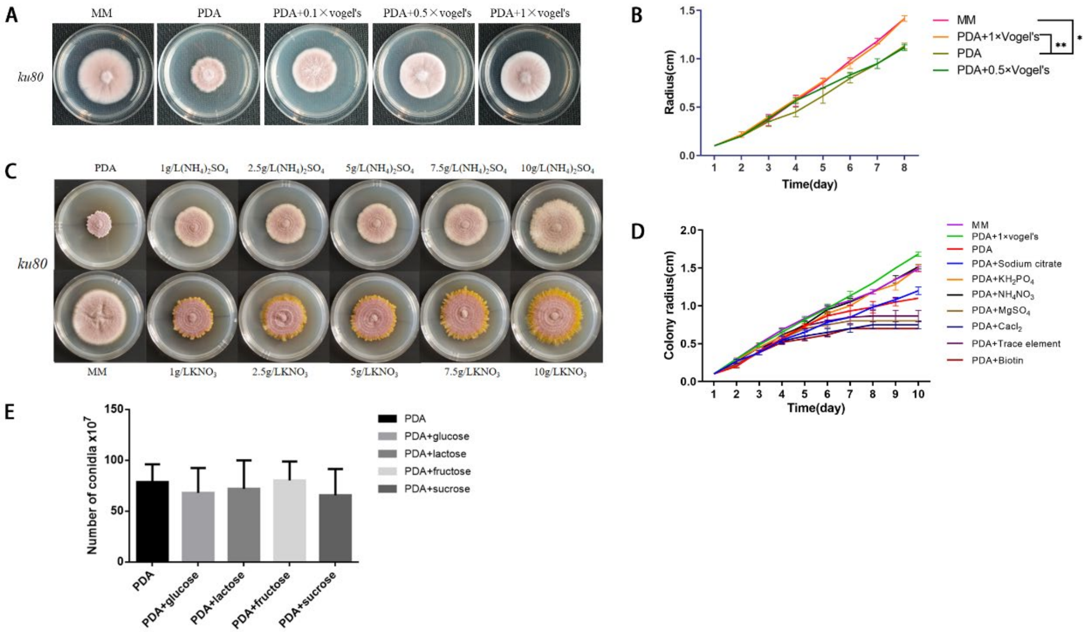

Figure S1. Growth and conidiation of *P. lavendulum* in various conditions. (A) The colony morphology of *ku80* strain on MM, PDA, and PDA with different concentrations of Vogel’s. (B) The growth curve of the *ku8*0 strain on MM, PDA, and PDA with different concentrations of Vogel’s. (C) Comparison of colony morphology of *ku80* Strain on MM, PDA, and PDA with different concentrations KNO_3 or $(NH_4)_2SO_4$. (D) Growth curve of *ku80* strain on MM, PDA, and PDA with MM components. (E) Conidiation comparison of *ku80* strain on PDA and PDA with four carbon sources.

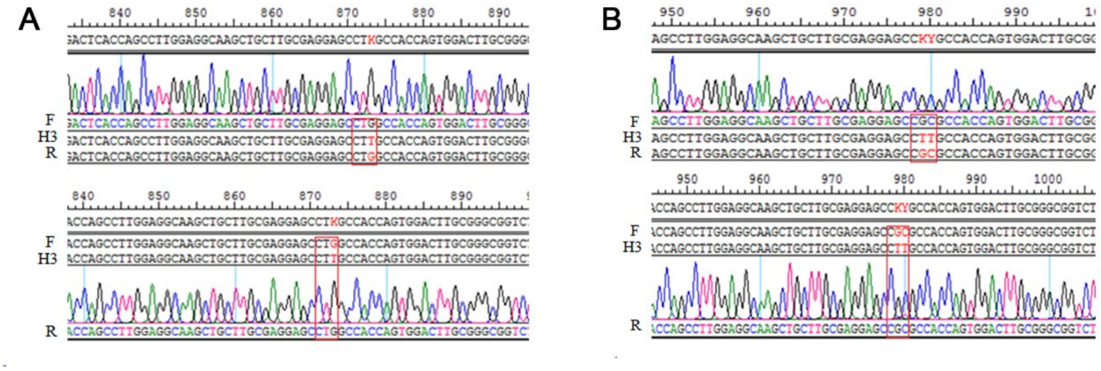

Figure S2. Sequencing H3 genes to confirmation of H3K14R and H3K14Q mutation. (A) CTT mutated to CTG, resulting in H3K14Q mutation. (B) CTT mutated to CGC, resulting in H3K14R mutation. F and R, a paired sequence of each mutant; H3, the wild-type H3 sequence.

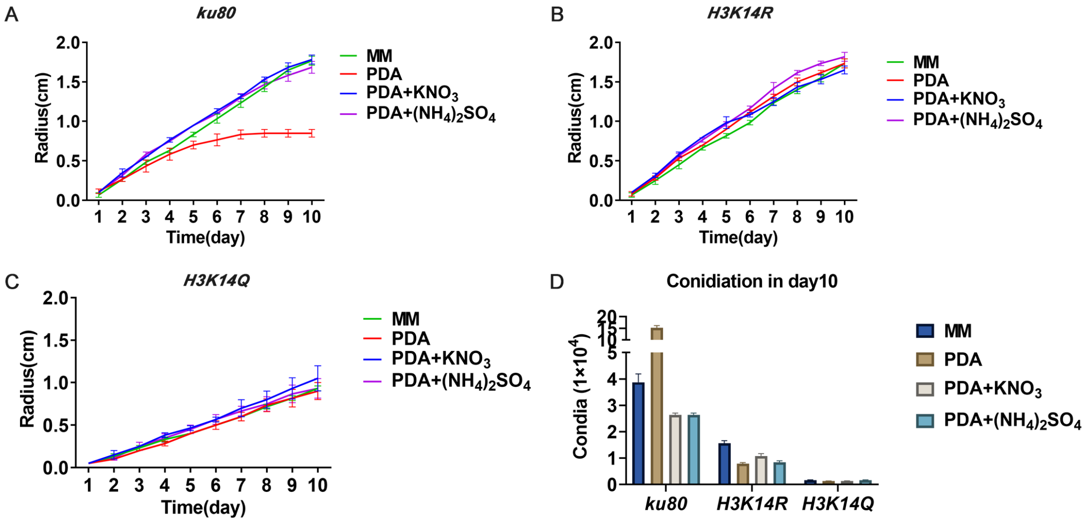

Figure S3. Growth and conidiation of *ku80* and two mutants on four different media. (A-C) Growth comparison of *ku80*, histone *H3K14R* and *H3K14Q* Mutants on four mediums: MM, PDA, PDA+KNO_3, and PDA+$(NH_4)_2SO_4$. (E) Conidiation comparison of *ku80 and* histone *H3K14R* and *H3K14Q* mutants on the above four mediums.

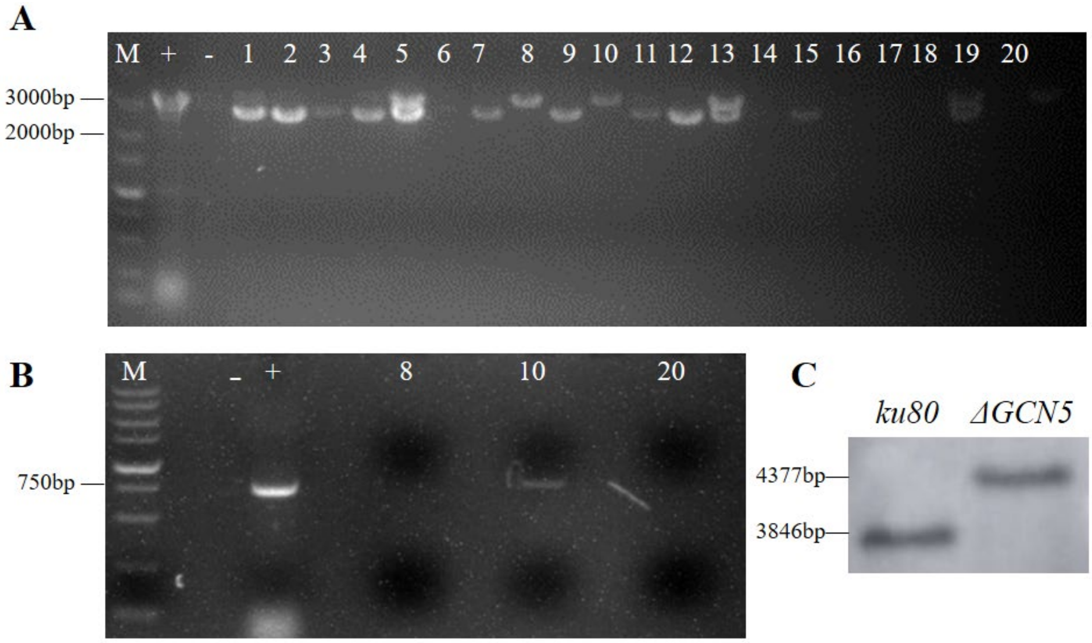

Figure S4. *Plgcn5* knockout validation. (A) PCR screening of *Plgcn*5 knockout strains. (B) PCR validation of random insertion of T-DNA in *ΔPlgcn5* knockout strains. (C) *ΔPlgcn5* knockout strain validated by Southern blot.

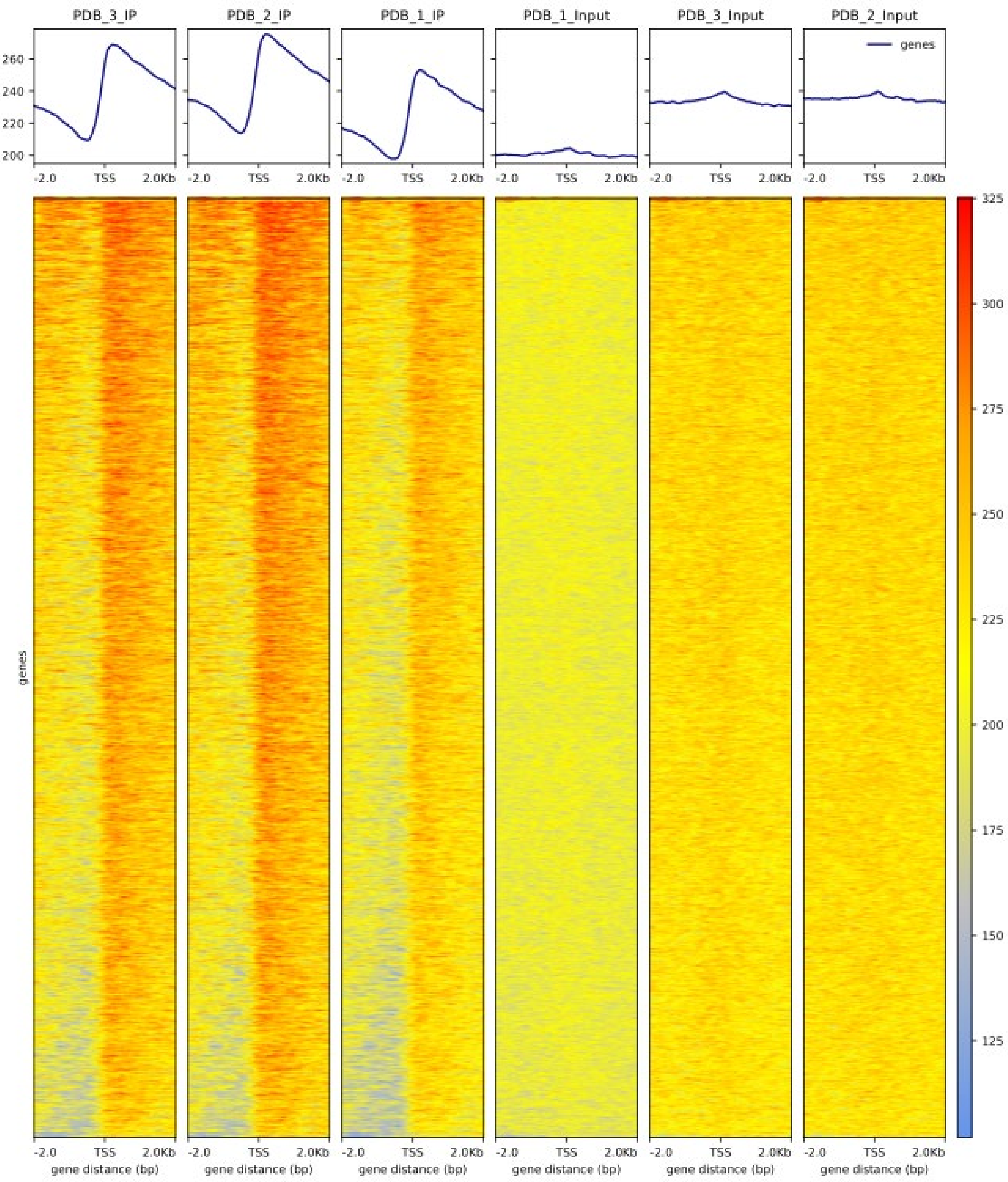

Figure S5. The heatmap of peak distribution around the TSS in samples cultured in PDB media.

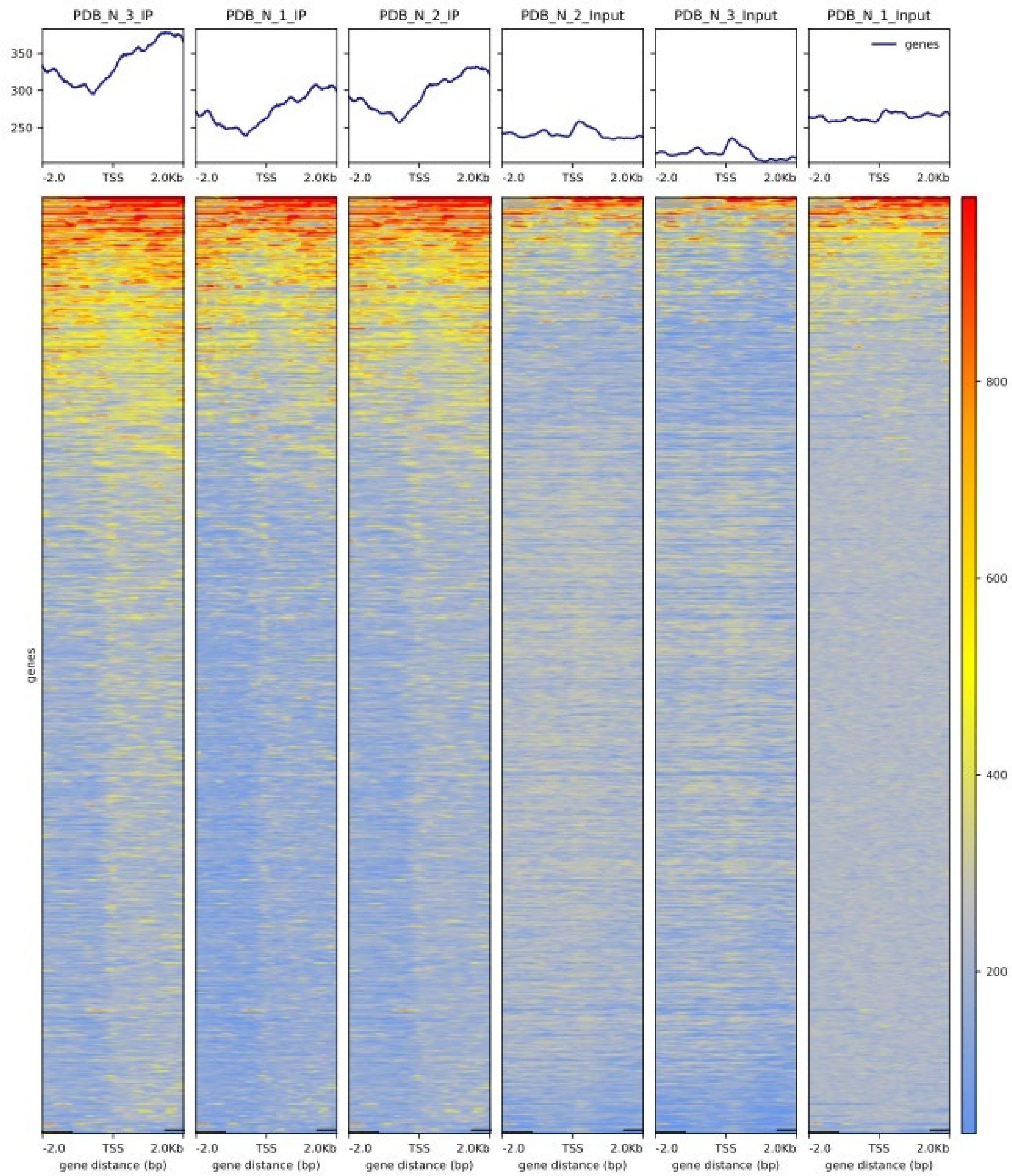

Figure S6. The heatmap of peak distribution around the TSS in samples cultured in PDB plus ammonium sulfate media.

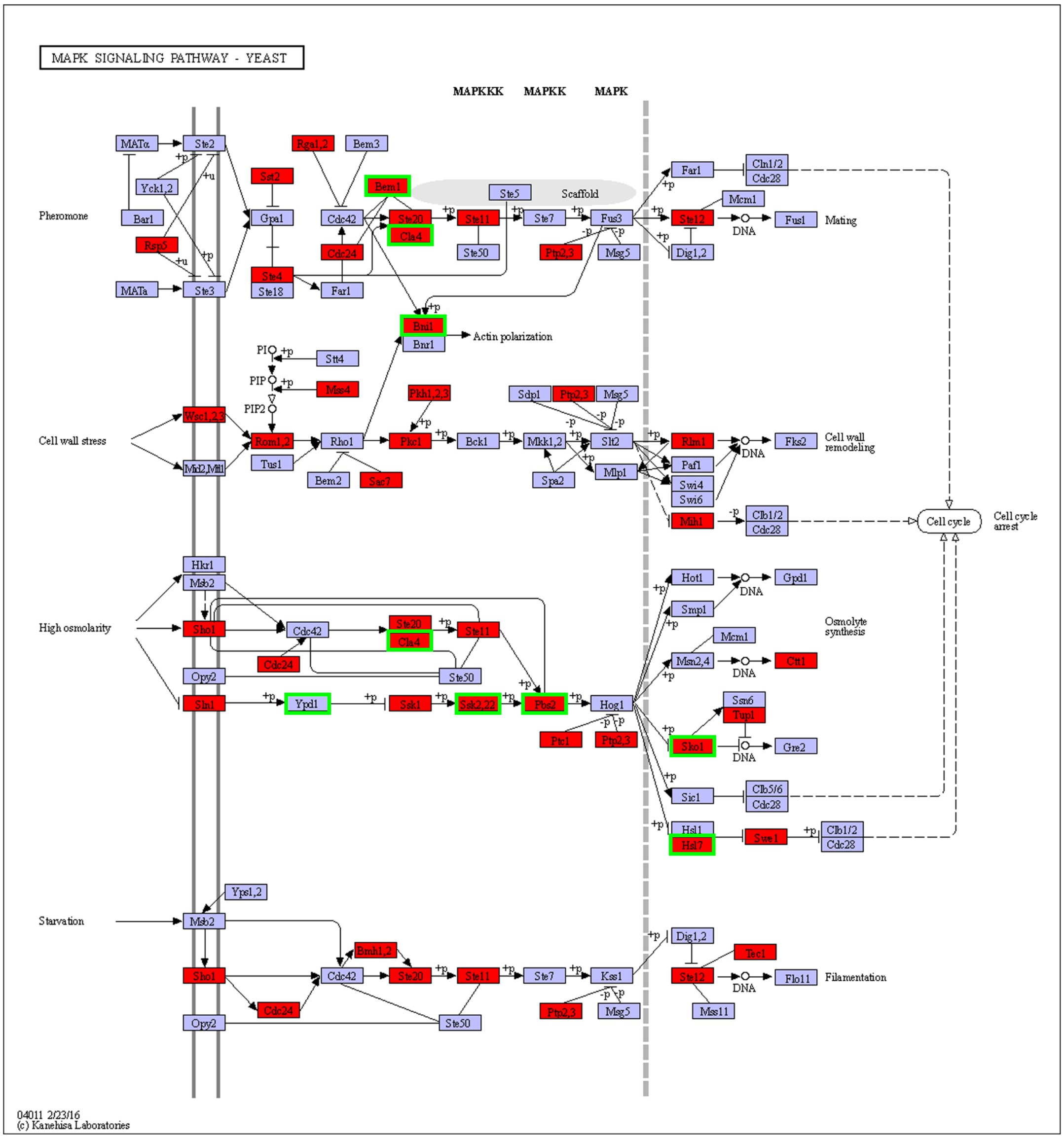

Figure S7. H3K14ac is enriched in the MAPK pathway. The genes in the red boxes were enriched in H3K14ac in the samples cultured in the PDB media. The genes in the green-border box were enriched in H3K14ac in the samples cultured in PDB plus ammonium sulphate media.
